# A uretheral stone causing urinary obstruction in a paediatric patient

**DOI:** 10.1016/j.eucr.2024.102650

**Published:** 2024-01-06

**Authors:** Mansoor Ahmed, Murad Habib, Huma Memon, Rafi Raz Ahmad, Fatima Rasheed, Muhammad Amjad Chaudhary

**Affiliations:** Department of Paediatric Surgery, The Children's Hospital, Pakistan Institute of Medical Sciences, Shaheed Zulfiqar Ali Bhutto Medical University, Islamabad, Pakistan

**Keywords:** Urethral stone, Paediatric surgery, Urology

## Abstract

Urinary retention in pediatric patients is an uncommon presentation, particularly when attributed to urethral stones. We present the case of an 8 year old child who experienced acute urinary retention due to a urethral stone, an infrequent occurrence in this age group. Initial assessment revealed signs of obstructive voiding, prompting further investigation. Imaging studies confirmed the presence of a urethral stone causing complete obstruction. Management involved a multidisciplinary approach, incorporating surgical intervention. This case report underlines the need for awareness about the rare occurrence of urethral stones in pediatric age group which must be kept in mind while treating children.

## 1 Introduction

The incidence of urethral stone is less than 0.3 % and are twenty times less common in children. Urinary retention secondary to a urethral stone in pediatric patients is a rare and challenging clinical scenario, often requiring a meticulous diagnostic approach to identify the underlying causes.^1^ Urethral stones, while infrequently encountered in the pediatric population, can pose significant complications, including obstructive urinary retention. This case report aims to shed light on a distinctive instance where a urethral stone precipitated urinary retention in a child. The rarity of this presentation emphasizes the importance of recognizing unusual etiologies in the assessment of pediatric urinary symptoms. By detailing the clinical course, diagnostic challenges, and management strategies in this specific case, we contribute to the growing body of knowledge surrounding pediatric urological conditions, ultimately enhancing our understanding of diverse clinical presentations in this vulnerable population.^2^

## 2 Case presentation

**Fig. 1:**
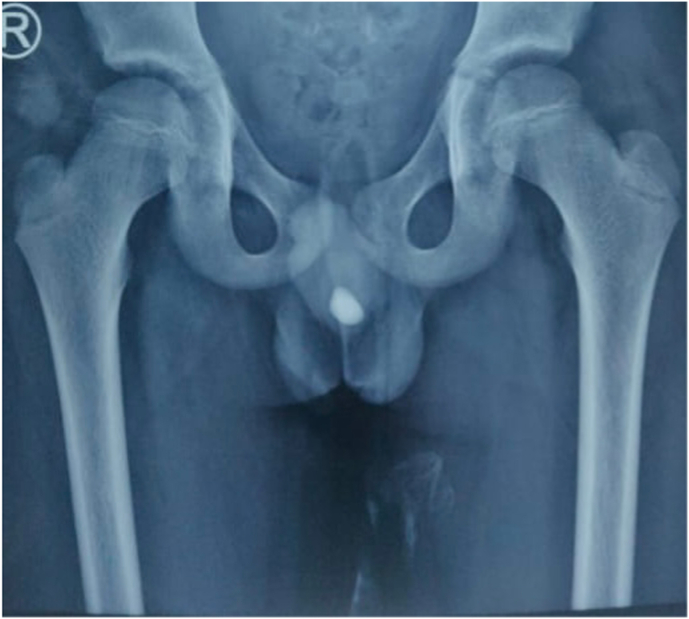
Radiograph showing a urethral stone.

**Fig. 2:**
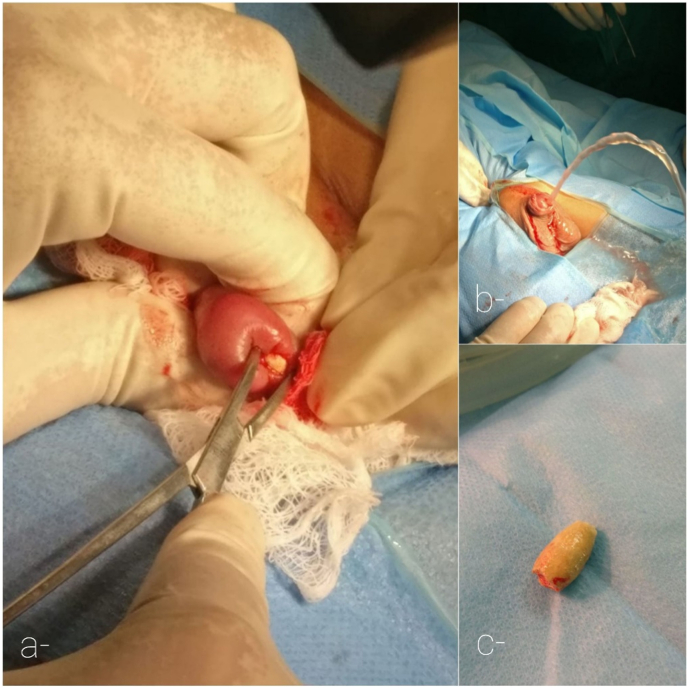
2a- Meatotomy being performed 2b- Patient passing urine after retrieval 2c- Uretheral stone.

A 8-year-old boy was presented to our emergency with the history of urinary retention from the last 20 hours. He had multiple visits to local clinic for increased urinary frequency, dysuria and urinary obstruction on and off, where he was prescribed oral antibiotics. Patient had no family history of stone disease. On arrival to the emergency department the patient was in agony. On examination, the abdomen was soft but distended and bladder was palpable. He was circumcised. The glans and external urinary meatus were tender to touch with redness underneath; a small stone was palpated at the base of the penis. Patient was resuscitated and intravenous antibiotics and analgesics were administered. Radiograph of the kidney, ureter, bladder was obtained which showed a urethral stone (Fig. 1). The emergency physician tried per-urethral catheterization but was unsuccessful. Patient was shifted to the operating room where Suprapubic catheterization was performed to relieve the urinary obstruction. Urine was sent for culture sensitivity and biochemical analysis. Patient was admitted to pediatric surgery ward. Under the general anesthesia, position of the stone was confirmed at the base of the penis, the urethra was then lubricated and stone was milked to the distal urethra where a meatotomy was performed and the stone was retrieved (Fig. 2).

Following the retrieval patient passed the urine. Supapubic catheter was removed and a catheter per-urethra was placed. Patient had uncomplicated course in the post-operative period and was discharged home on second post-operative day and was called for follow up. His stone analysis revealed a calcium oxalate stone.

## 3 Discussion

Urethral stones when presented are a cause of increased morbidity in children. The present case underscores the atypical occurrence of urinary retention in a 6-year-old boy attributed to a urethral stone, a condition rarely encountered in the pediatric population. The successful resolution of the patient's symptoms following the removal of the urethral stone highlights the importance of prompt diagnosis and intervention in such cases.^3^

The clinical presentation of urinary retention in pediatric patients can pose a diagnostic challenge due to its association with various etiologies. In this instance, the initial obstructive voiding symptoms raised suspicion, prompting a comprehensive evaluation that led to the identification of a urethral stone. This emphasizes the need for a high index of suspicion and a thorough diagnostic workup when faced with uncommon presentations of urinary symptoms in children.^4^

The successful management of this case involved a multidisciplinary approach. Conservative measures, including hydration and analgesia, were initially employed to stabilize the patient. However, given the persistent nature of the urinary retention and the identification of the urethral stone on imaging, surgical intervention became necessary. The timely removal of the urethral stone proved to be crucial in alleviating the obstruction and restoring normal urinary function.^5^

The age of the patient adds a layer of complexity to the case, as urethral stones are more commonly associated with adults. The underlying etiology in pediatric cases may differ, necessitating a tailored approach in diagnosis and management. Awareness of such atypical presentations is essential for clinicians to avoid delays in treatment and optimize outcomes.

While urethral stones in children are rare, this case report contributes to the existing literature by providing insights into the diagnostic challenges and successful management of this unique scenario. Continued documentation and analysis of similar cases will further enrich our understanding of pediatric urological conditions, ultimately guiding clinicians in delivering effective and timely care.

## 4 Conclusion

Urethral stone is rare in paediatric patients and if presents with acute urinary obstruction, multiple attempts to pass urethral catheter should be discouraged and stone should be removed surgically.

## Funding

No funding received for this research.

## Ethical approval

This study was exempted by the ethical review board of Pakistan Institute of Medical Sciences Islamabad.

## Informed Consent

Patient's attendant (father) provided informed consent prior to study participation.

## Data availability

The data that support the findings of this study are available from the corresponding author upon request to corresponding author.

## CRediT authorship contribution statement

**Mansoor Ahmed:** Conceptualization. **Murad Habib:** Conceptualization. **Huma Memon:** Data curation. **Rafi Raza:** Data curation. **Fatima Rasheed:** Visualization. **Muhammad Amjad Chaudhary:** Supervision.

## Declaration of competing interest

The authors have no conflicts of interest relevant to this article to disclose.
